# Survey data of public in Sindh Pakistan regarding willingness to accept COVID-19 vaccination

**DOI:** 10.1371/journal.pone.0270900

**Published:** 2022-08-25

**Authors:** Narendar Kumar, Syed Azhar Syed Sulaiman, Furqan Khurshid Hashmi, Ali Qureshi, Muhammad Shaib, Shoaib Alam, Mujahid Hussain

**Affiliations:** 1 Discipline of Clinical Pharmacy, School of Pharmaceutical Sciences, Universiti Sains Malaysia, George, Pulau Penang, Malaysia; 2 Department of Pharmacy Practice, Faculty of Pharmacy, University of Sindh, Jamshoro, Pakistan; 3 University College of Pharmacy, University of the Punjab, Allama Iqbal Campus, Lahore, Pakistan; 4 Department of Pharmaceutics, Faculty of Pharmacy, University of Sindh, Jamshoro, Pakistan; 5 Sindh Government Hospital, Karachi, Pakistan; 6 Department of Pharmacy Services, The Indus Hospital and Health Network, Karachi, Pakistan; Shaheed Montarma Benazir Bhutto Medical University, PAKISTAN

## Abstract

**Objective:**

The COVID-19 pandemic has badly affected the world with its devastating effects, including Sindh, Pakistan. A massive vaccination campaign against COVID-19 is considered one of the effective ways to curtail the spread of the disease. However, the acceptability of the COVID-19 vaccine is based on the general population’s knowledge, attitude, perception and willingness for vaccination. Therefore, a survey among the public in Sindh, Pakistan, was done to evaluate their knowledge, attitude, perception and willingness to accept COVID-19 vaccination.

**Method:**

The online survey was conducted among the residents of Sindh, Pakistan, in July 2021 through a survey tool designed using Google Forms and sent to the population through various social media.

**Results:**

Of 926 study participants, 59.0% were male, and 68.6% were aged between 18 and 31 years. Higher percentages of responses were recorded from the Hyderabad division (37.5%), and 60.0% of respondents were graduates, with 34.8% of them in the private sector. The results showed that 36.4% of respondents had good knowledge, and 30.3% had a positive attitude toward COVID-19 vaccination. Almost 77% of respondents perceived that everyone should get vaccinated in the country and those health care workers on priority. A majority (80.8%) of respondents were willing to accept COVID-19 vaccination.

**Conclusion:**

Despite having insufficient knowledge and a low percentage of positive attitude public in Sindh are willing to be vaccinated. Based on this finding, more effort has to be done to promote vaccination among the public, especially among the less educated population.

## Introduction

The spread of COVID-19 around the globe led to the accelerated development of vaccines to achieve immunity against the virus. Besides the inclination in the number of positive cases worldwide, various vaccines have now been approved by health regulatory authorities. They are being administered to the population throughout the world to decline the spread of COVID-19. These vaccines play a vital role in decreasing the mortalities and morbidities associated with the disease [1, 2]. However, the satisfaction and acceptability of COVID-19 vaccines are solely based on the level of knowledge, attitude and perception of the public [3]. The chances of hospitalization declined by 17 folds among the vaccinated subjects who received complete COVID-19 vaccination [4].

On February 26, 2020, the first positive case of COVID-19 was reported in Sindh, Pakistan. Since that time, COVID-19 is continuously producing its devastating effects around the globe, including in Sindh, Pakistan. As of July 8, 2021, the province of Sindh has recorded as high as 344,223 positive cases and 5566 deaths in the country [5].

The war against the COVID-19 pandemic is going on [6]; scientists have developed numerous vaccines against the disease. Health authorities have also prioritized the highly vulnerable group for vaccination, such as health care workers and the geriatric population in countries throughout the world [7]. According to the virology experts, the COVID-19 pandemic will consistently increase till the availability of vaccines for underdeveloped countries [8].

Literature published earlier from Africa, Europe, France, and India has revealed that the knowledge and attitudes of the general public towards COVID-19 vaccines ranged between 31% and 86%, respectively [9–12].

The public’s knowledge, attitude, and perception of COVID-19 vaccination are essential factors for its acceptability. Poor knowledge, inappropriate attitude and wrong perception of the COVID-19 vaccine are grave concerns worldwide. Besides the efficient means of curtailing the spread of the virus with standard operating procedures (SOPs) [13], it is equally essential to vaccinate the majority of the population, particularly the high-risk groups [14]. Therefore, the research study is designed to determine the levels of knowledge, attitude, perception and predictors of willingness to accept the COVID-19 vaccine in the general population of Sindh, Pakistan.

## Methods

### Study design and sampling procedure

This research study was conducted in Province Sindh, geographically in the southeastern part of Pakistan [15]. Based on the population, it is the second-largest province of Pakistan, consisting of 7 divisions; Bhanbhore, Hyderabad, Karachi, Larkana, Mirpurkhas, Shaheed Benazirabad and Sukkur [16].

A cross-sectional study using a web-based survey was conducted in the population of Sindh, Pakistan, in July 2021. Firstly, the survey instrument was adapted from the previous studies [17–19]. Then, the initial draft of the survey was designed in English and Urdu using Google Forms and was shared with a team of three academic experts in the research area. After their consultation and consensus, the final version of the survey was designed and underwent a pilot study consisting of 50 respondents for content validation and reliability. The internal reliability of the pilot study was tested through Cronbach’s alpha coefficient using SPSS v22. The value of Cronbach’s alpha for the survey was 0.691. The survey instrument was designed using Google Forms with an appended consent statement, and the link was promoted through various social media contacts and groups, including Facebook, WhatsApp and Telegram. The respondents were also encouraged to share the link with their contacts, and so on. The respondents received information about the research study and informed consent by clicking the provided link. Once they agreed to participate in the online survey, they had to fill in the demographic information. Then, several questions based on their knowledge, attitude, perception and willingness to accept the COVID-19 vaccine appeared which were to be responded, sequentially. The online survey was open to; 1) all the residents of the province of Sindh, Pakistan, 2) respondents aged ≥18 years, and 3) those who had internet facilities and could understand either English or Urdu. The participants could fill out and submit it using a computer or cell phone. The participants could fill out the survey in around 5–7 minutes. Initially, a sum of 1044 responses was recorded during the study period. Of them, 926 respondents had filled out the survey altogether, which gave a response rate of 88.69%.

### Measures

The online survey included information based on research background, motivations, confidentiality statement, voluntary contribution, and items based on their knowledge, attitude, perception and willingness to accept vaccination against COVID-19. The questionnaire contained 7 questions on respondents’ demographic information, including; gender, age, marital status, residential area, division of residence, academic qualification and profession. There were 2 questions regarding the personal history of COVID-19 infection and any death reported from family members, relatives and friends due to COVID-19 infection. And then, there were 17 items regarding the knowledge, attitudes, perception and willingness toward the acceptability of the COVID-19 vaccine.

### Knowledge, attitudes, perception and willingness to accept COVID-19 vaccine

The knowledge portion consisted of seven questions that led to an awareness of the COVID-19 vaccine, its effectiveness and spectrum of efficacy, side reactions, and degree of protection after vaccination. The answers were either "No" or "Yes" assigned dichotomous responses. 1 point was given for each correct answer and 0 points for each wrong answer. The overall outcome of the knowledge score was measured by summing all the values. Consequently, respondents having a knowledge score higher than the mean were considered to have "Good Knowledge", those having equaled to mean were considered to have "Average Knowledge", and those having less than the mean were considered to have "Poor Knowledge".

Respondents’ attitudes were measured by four statements based on their belief in government-provided vaccinations, encouraging others to vaccination and the spread of COVID-19 after vaccination. All statements were rated on a 5-point Likert scale, including Strongly disagree (1-point), Disagree (2-points), Neutral (3-points), Agree (4-points) and Strongly agree (5-points). The total attitude score was measured by summing up all the values. Accordingly, the respondent with attitude scores higher than the mean, equal to the mean and less than the mean were counted to reflect positive, neutral, and negative attitudes, respectively.

Three statements assessed respondents’ perception: the possibility of side effects due to the COVID-19 vaccine, control of COVID-19 spread without vaccination, and chances of COVID-19 after vaccination. Every statement was assigned five options, including Strongly disagree (1-point), Disagree (2-points), Neutral (3-points), Agree (4-points) and Strongly agree (5-points). Two additional statements regarding respondents’ perceptions were: "According to you, who should get vaccinated?" and "What do you think, who should get vaccinated on priority?"

A single choice statement analyzed respondents’ willingness to accept the COVID-19 vaccine; "No or Yes".

### Statistical analysis

Data was generated using Statistical Package for Social Sciences version 22.0 (SPSS v.22 from IBM Corporation, Armonk, NY). Continuous variables were expressed as Means ± standard deviations. However, categorical variables were expressed in frequencies and percentages. A comparison of knowledge and attitude levels was made through Chi-square tests for different groups of variables. Kruskal Wallis tests and Mann Whitney U tests were applied to compare the mean knowledge and attitude scores among different group variables. All *p-values* less than or equal to 0.05 was considered significant. Multinomial logistic regression was performed to predict the factors responsible for accepting the COVID-19 vaccine among respondents.

### Ethical approval

This research was ethically approved by the Institutional Bioethics Committee (IBC), University of Sindh, Jamshoro, Pakistan (Ref. No. ORIC/SU/845). Anonymity and confidentially were highly maintained during the process of data collection.

## Results

Out of 926 respondents, 59.0% were male, and around 2/3^rd^ respondents, 68.6%, were lying in the age group between 18 and 31 years. The average age of the respondents (mean ± SD) was 28.9±8.9. More than half, 59.9%, of respondents were single, and belonged to urban areas (60.4%). Higher response percentages were recorded from Hyderabad, Mirpurkhas, and Karachi divisions as 37.5%, 17.8% and 16.6%, respectively. Approximately 60.0% of respondents held a graduation degree, and around one third (34.8%) were doing a private job. Only 13.5% of respondents reported getting an infection of COVID-19, and 27.6% of respondents had lost their family members/relatives/friends due to COVID-19 infection (Table 1).

**Table 1:** Basic demographic data for respondents in Sindh Pakistan.

Respondents’ Characteristics		Frequency	Percentage
**Gender**	Female	380	41.0%
	Male	546	59.0%
**Age Groups**	18–31 years	637	68.8%
	32–50 years	250	27.0%
	> 50 years	39	4.2%
**Marital Status**	Single	555	59.9%
	Married	363	39.2%
	Widowed/Divorced	8	0.9%
**Residential Area**	Rural	367	39.6%
	Urban	559	60.4%
**Divisions**	Bhanbhore	49	5.3%
	Hyderabad	347	37.5%
	Karachi	154	16.6%
	Larkana	63	6.8%
	Mirpurkhas	165	17.8%
	Sukkur	65	7.0%
	Shaheed Benazir Abad	83	9.0%
**Academic Qualification**	Primary/Matric	9	0.9%
	Intermediate	104	11.2%
	Diploma	11	1.2%
	Graduate	556	60.0%
	Post-graduate	246	26.6%
**Profession**	Government Job	151	16.3%
	Private Job	322	34.9%
	Business	56	6.0%
	Retired	7	0.8%
	Daily Wager/Labor	9	0.9%
	Student	295	31.9%
	Housewife	36	3.9%
	Unemployed	50	5.4%
Did you ever get infected with COVID-19?	No	801	86.5%
	Yes	125	13.5%
Did anyone from your family members/relatives/friends died of COVID-19?	No	670	72.4%
	Yes	256	27.6%

### Respondents’ knowledge for COVID-19 vaccination

The average knowledge score (mean ± SD) was 5.03 ± 1.11. A large majority of respondents, 93.4% were familiar with the COVID-19 vaccine, 83.4% were aware of the efficacy of the COVID-19 vaccine, and 46.1% had believed in the vaccine’s effectiveness against different COVID-19 variants. Around 85.4% of respondents thought that an overdose of the vaccine could be dangerous for life, 68.4% believed that the COVID-19 vaccine could cause allergic reactions, 27.3% thought that the COVID-19 vaccine might be responsible for autoimmune diseases, 53.1% had believed that despite being vaccinated they could be the source of spreading COVID-19. Furthermore, about 1/3^rd^ of respondents, 36.4%, had good knowledge of the COVID-19 vaccine, as presented in Table 2.

**Table 2:** Knowledge of respondents for COVID-19 vaccination.

Questions		Frequency	Percentage
Are you aware of COVID-19 vaccine?	No	61	6.6%
	Yes	865	93.4%
Do you think that COVID-19 vaccine is effective?	No	154	16.6%
	Yes	772	83.4%
Are available COVID-19 vaccines effective against all COVID-19 variants?	No	499	53.9%
	Yes	427	46.1%
Is it really dangerous to use vaccine in excess?	No	133	14.4%
	Yes	793	85.6%
Does vaccination against COVID-19 may cause allergic reactions?	No	293	31.6%
	Yes	633	68.4%
Does vaccination increase autoimmune diseases?	No	673	72.7%
	Yes	253	27.3%
Can you be the source of spread COVID-19 despite being vaccinated?	No	434	46.9%
	Yes	492	53.1%
Status of knowledge towards COVID-19 vaccine	Poor	74	8.0%
	Average	515	55.6%
	Good	337	36.4%

### Respondents’ attitude for COVID-19 vaccination

The average (mean ± SD) score for attitude for COVID-19 vaccination was 18.61 ± 3.82. Nearly half, 48.6% of the respondents believed that the vaccine provided by the Government of Pakistan was very safe, 51.0% agreed that everyone should get vaccinated against COVID-19, and 31.9% of respondents strongly agreed to recommend vaccination of their family members, relatives, and friends against COVID-19. Approximately 40% of respondents strongly agreed that the incidences of COVID-19 cannot be reduced without vaccination. Overall, 30.3% of surveyed respondents had a positive attitude toward the COVID-19 vaccine, as described in Table 3.

**Table 3:** Attitude of respondents for COVID-19 vaccine.

Statements	Response	Frequency	Percentage
The COVID-19 vaccine provided by Government of Pakistan is very safe.	Strongly disagree	32	3.5%
	Disagree	61	6.6%
	Neutral	330	35.6%
	Agree	450	48.6%
	Strongly agree	53	5.7%
It is necessary that everyone should get vaccinated against COVID-19	Strongly disagree	41	4.4%
	Disagree	48	5.2%
	Neutral	118	12.7%
	Agree	472	51.0%
	Strongly agree	247	26.7%
You should recommend COVID-19 vaccine to your family, relatives and friends	Strongly disagree	38	4.1%
	Disagree	45	4.9%
	Neutral	104	11.2%
	Agree	444	47.9%
	Strongly agree	295	31.9%
It is impossible to reduce the incidence of COVID-19 without vaccination.	Strongly disagree	68	7.3%
	Disagree	157	17.0%
	Neutral	159	17.2%
	Agree	372	40.2%
	Strongly agree	170	18.4%
Status of Attitude towards COVID-19 vaccine	Negative	148	16.0%
	Average	497	53.7%
	Positive	281	30.3%

### Respondents’ perception for COVID-19 vaccine

Regarding the perception towards COVID-19 vaccination, nearly one-third (33.1%) of respondents believed that the COVID-19 vaccine could cause serious side effects, 42.2% answered that COVID-19 could be eradicated without a vaccine if everyone adopts precautionary measures, and 19.4% perceived that there was a low risk of COVID-19 after being vaccinated. Around 3/4^th^ of respondents, 77.0% had perceived that everyone in the country should get vaccinated, and 77.6% perceived that the health care professionals should be vaccinated on priority (Table 4).

**Table 4:** Perception of respondents for COVID-19 vaccination.

Statements	Response	Frequency	Percentage
The COVID-19 vaccine may have serious side effects.	Strongly disagree	32	3.5%
	Disagree	214	23.0%
	Neutral	300	32.4%
	Agree	306	33.1%
	Strongly agree	74	8.0%
If everyone adopts all precautionary measure, then COVID-19 pandemic can be eliminated without a vaccine	Strongly disagree	74	8.0%
	Disagree	186	20.1%
	Neutral	166	17.9%
	Agree	391	42.2%
	Strongly agree	109	11.8%
There is a low risk of COVID-19 infection after being vaccinated.	Strongly disagree	105	11.3%
	Disagree	392	42.3%
	Neutral	238	25.7%
	Agree	180	19.4%
	Strongly agree	11	1.2%
Who should get vaccinated?	People without COVID-19 exposure	107	11.6%
	People with COVID-19 exposure	79	8.5%
	People who have recently recovered from COVID-19	27	2.9%
	Everyone in the country	713	77.0%
Who should get vaccinated on priority?	Health care workers	719	77.6%
	Government/Private employees	10	1.1%
	Students and Teachers	21	2.3%
	Labor/Daily wager	29	3.1%
	General Public	147	15.9%

### Respondents’ willingness to accept COVID-19 vaccination

**Fig 1:**
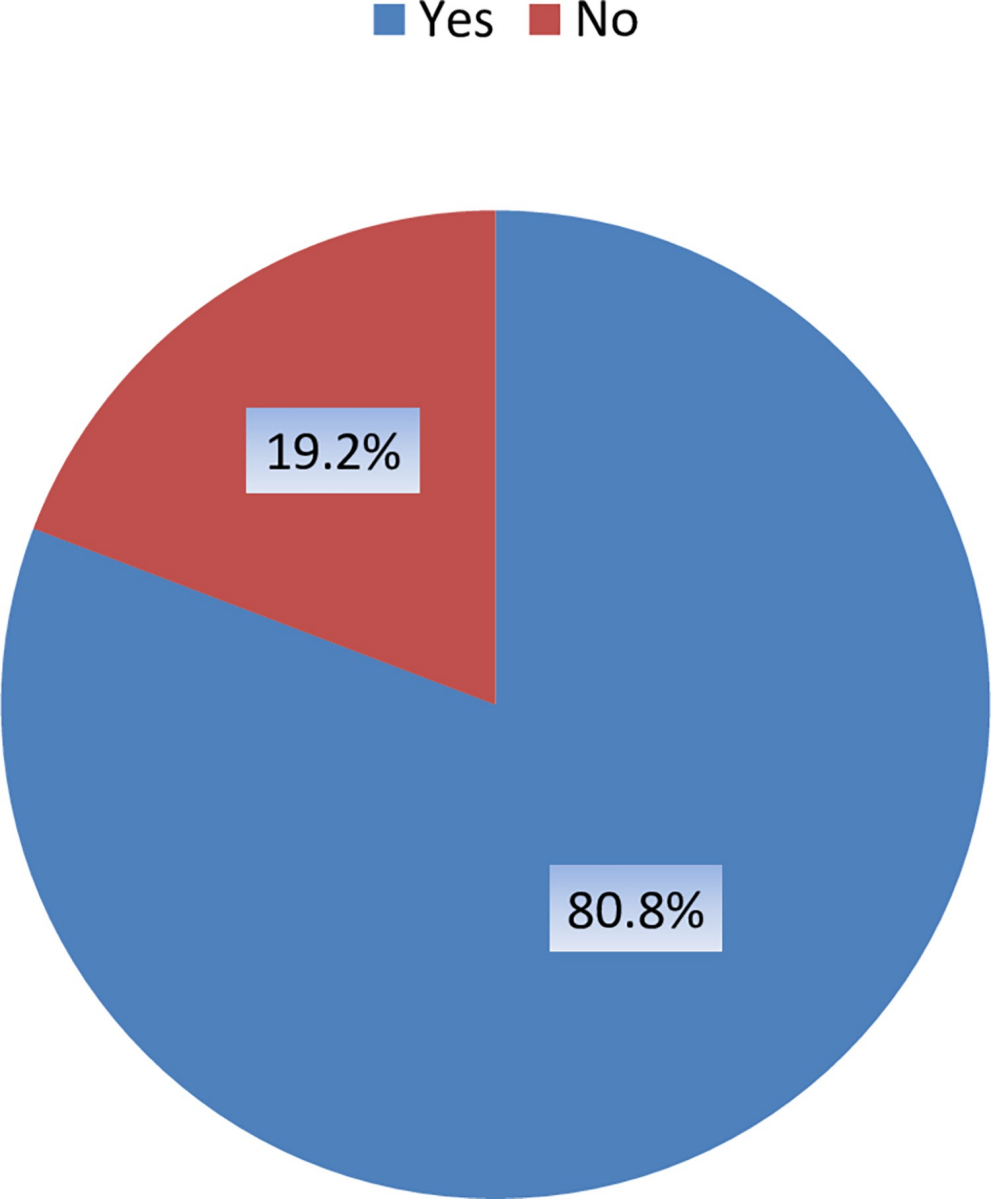
Respondents’ willingness to accept COVID-19 vaccination.

Regarding the willingness to accept the COVID-19 vaccine, 748 (80.8%) respondents were very willing to be vaccinated, offered by the government without any hesitation. Whereas only 178 (19.2%) were reluctant to get vaccinated against COVID-19, as shown in Fig 1.

### Predictors of willingness to accept COVID-19 vaccine

Table 5 presents the predictors of respondents’ willingness to accept the COVID-19 vaccine. Multinomial logistic regression was performed to predict the factors associated with COVID-19 vaccine acceptability. Accordingly, variables such as gender, age groups, division, academic qualification, and profession were analyzed using linear regression.

**Table 5:** Predictors of respondent’s willingness to accept COVID-19 vaccination.

	Will you get vaccinate against COVID-19?			
Variables	Yes	No	OR (95% CI)	*p-value*
**Gender**				
Female	303	77	1.00	
Male	445	101	0.94 (0.6–1.4)	0.79
**Age Groups**				
18–31 years	504	133	1.00	
32–50 years	210	40	1.3 (0.78–2.1)	0.308
> 50 years	34	5	2.0 (0.62–6.9)	0.232
**Divisions**				
Bhanbhore	44	5	1.00	
Hyderabad	265	82	0.33 (0.12–0.9)	0.031*
Karachi	132	22	0.61 (0.2–1.8)	0.372
Larkana	47	16	0.32 (0.1–0.98)	0.046*
Mirpurkhas	140	25	0.64 (0.2–1.8)	0.400
Sukkur	52	13	0.42 (0.13–1.3)	0.137
Shaheed Benazir Abad	68	15	0.41 (0.13–1.25)	0.119
**Academic Qualification**				
Primary/Matriculation	4	5	1.00	
Intermediate	81	23	5.06(1.14–22)	0.032*
Diploma	11	0		0.998
Graduation	451	105	7.11 (1.6–29.9)	0.008*
Post-graduation and others	201	45	5.19 (1.1–22.8)	0.029*
**Profession**				
Government Job	136	15	1.00	
Private Job	255	67	0.41 (0.2–0.79)	0.008*
Business	37	19	0.17 (0.07–0.4)	0.001*
Retired	6	1	0.36(0.03–4.2)	0.421
Daily Wager/Labor	9	0		0.999
Student	240	55	0.61 (0.29–1.2)	0.201
Housewife	27	9	0.38 (0.13–1.13)	0.083
Unemployed	38	12	0.37 (0.48–0.95)	0.040*

Multinomial logistic regression determining the predictors of willingness to accept COVID-19 vaccine

*p-value <0.05.

The independent predictors for intention to accept the COVID-19 vaccine include age > 50 years with an Odd Ratio OR: 2.0 [95% CI, 0.62–6.9] and having graduation degrees with OR: 7.113 [95% CI, 1.6–29.9]. COVID-19 vaccine acceptance was twice more likely among respondents aged > 50 years OR: 2.0 [95% CI, 0.62–6.9] than respondents aged 18–31 years. Additionally, having a graduation degree was another contributing factor to accepting COVID-19 vaccine, which was approximately seven times more than primary and matriculation respondents OR: 7.11 [95% CI, 1.6–29.9].

### Inferential analysis of status of knowledge and attitude versus respondents’ characteristics

The associations of the status of knowledge and attitudes with respondents’ characteristics were determined by applying a chi-square test. Regarding the status of knowledge, there was no significant difference among the gender and age groups. Nevertheless, there were substantial differences when comparing the status of knowledge within divisions (*p* = 0.011), academic qualification (*p* = 0.001) and profession (*p* = 0.035). Similarly, while comparing attitude status, there was no significant difference between gender and age groups. However, there was a marked difference with different groups of divisions (*p* = 0.043), academic qualification (*p* = 0.007) and profession (*p* = 0.012), as shown in Table 6.

**Table 6:** Inferential analysis of status for knowledge and attitude versus respondents’ characteristics.

Factors	Knowledge						Attitude					
	Poor	Average	Good	χ^2^	df	*p-value*	Negative	Average	Positive	χ^2^	df	*p-value*
**Gender**												
Female	30	208	142	0.265	2	0.876	63	199	118	0.453	2	0.797
Male	44	307	195				85	298	163			
**Age Groups (years)**												
18–31	59	355	223	5.589	4	0.232	108	350	179	6.769	4	0.149
32–50	12	139	99				37	127	86			
> 50	3	21	15				3	20	16			
**Divisions**												
Bhanbhore	5	30	14	25.936	12	0.011*	2	35	12	21.580	12	0.043*
Hyderabad	24	201	122				72	178	97			
Karachi	12	68	74				24	78	54			
Larkana	8	40	15				7	38	18			
Mirpurkhas	16	88	61				21	87	57			
Sukkur	6	44	15				9	33	23			
Shaheed Benazirabad	3	44	36				13	50	20			
**Academic Qualification**												
Primary/Matriculation	2	4	3	32.579	8	0.001*	4	4	1	21.103	8	0.007*
Intermediate	11	67	26				16	47	41			
Diploma	4	0	7				0	9	2			
Graduation	45	298	21				87	319	150			
Post-graduation and others	12	146	88				41	118	87			
**Profession**												
Government Job	6	94	51	24.929	14	0.035*	14	75	62	28.602	14	0.012*
Private Job	23	184	115				60	177	85			
Business	5	33	18				12	35	9			
Retired	1	3	3				0	6	1			
Daily Wager/Labour	2	7	0				0	6	3			
Student	28	153	114				47	153	95			
Housewife	1	15	20				6	23	7			
Unemployed	8	26	16				9	22	19			

Chi-square test giving comparison status of knowledge and attitude with group variables.

*p-value <0.05.

### Comparison of knowledge and attitude among different demographic groups

The average knowledge and attitude scores (mean ± SD) were determined for different variables, including gender, age group, marital, division, education and profession. There was no substantial difference in knowledge scores among different variable groups except among the respondents from various divisions (*p* = 0.013) (Table 5).

Kruskal Wallis tests and Mann- Whitney U was applied to compare the mean knowledge and attitude score among different groups of variables. There was a noteworthy difference (*p* = 0.013) in knowledge scores among the respondents from different divisions. Accordingly, the attitude score from variable including age groups (*p* = 0.006), division (*p* = 0.050), education (*p* = 0.012) were predominantly different within their groups (Table 7).

**Table 7:** Comparison of mean scores for knowledge and attitude among different group variables.

Factors	Knowledge		Attitude	
	Mean±SD	*p-value*	Mean±SD	*p-value*
**Gender**				
Female	5.04±1.02	0.753	14.83±2.86	0.571
Male	5.02±1.15		14.78±3.09	
**Age Groups (years)**				
18–31	4.98±1.12	0.109	14.67±2.85	0.006*
32–50	5.17±1.04		15.04±3.24	
> 50	5.03±1.07		15.48±3.46	
**Divisions**				
Bhanbhore	5.02±1.09	0.013*	15.22±2.35	0.050*
Hyderabad	5.01±1.07		14.46±3.12	
Karachi	5.19±1.04		14.94±3.23	
Larkana	4.67±1.13		14.93±2.46	
Mirpurkhas	5.00±1.21		15.18±2.90	
Sukkur	4.94±0.98		15.24±3.16	
Shaheed Benazirabad	5.19±1.04		14.55±2.66	
**Academic Qualification**				
Primary/Matric	4.67±1.58	0.100	12.00±3.64	0.012*
Intermediate	4.82±1.06		15.13±2.83	
Diploma	5.27±1.84		15.45±1.12	
Graduate	5.04±1.11		14.76±2.73	
Post-graduate	5.08±1.01		14.83±3.58	
**Profession**				
Government Job	5.04±0.97	0.080	15.30±2.88	0.061
Private Job	5.08±1.06		14.61±3.11	
Business	4.96±1.11		13.94±4.01	
Retired	5.00±1.41		14.85±1.21	
Daily Wager/Labour	4.11±0.78		16.11±2.02	
Student	5.00±1.18		14.86±2.77	
Housewife	5.33±0.98		14.27±2.53	
Unemployed	4.82±1.17		15.28±2.92	

Kruskal Wallis, Mann Whitney U tests determining the association of knowledge and attitude scores with different group variables

*p-value <0.05.

## Discussion

There is a unanimous consensus among world health experts that a massive global vaccination drive against COVID-19 is the ultimate solution to curtail this deadly pandemic. Infectious diseases may be prevented by the safe and effective use of vaccines [20]. However, delaying and denying vaccination has been a perplexing problem observed in the general population worldwide. Meanwhile, sufficient knowledge, appropriate attitude and positive perception of the COVID-19 vaccine may be essential to its acceptability [21]. Therefore, this research study was performed to assess the knowledge, attitude, and perception toward the COVID-19 vaccine in the population of Sindh, Pakistan. This assessment is crucial to assist the government authorities in educating the vulnerable groups about getting vaccinated throughout the country, mainly in the province of Sindh, which has recorded the highest number of COVID-19 cases in Pakistan. Our study discovered that only 36.4% and 30.3% of respondents had good knowledge and positive attitude towards COVID-19 vaccines, whereas 77.0% of respondents perceived that everyone should get vaccinated against the COVID-19 vaccine offered by Government.

Our study findings suggest that only 36.4% of respondents had good knowledge regarding the COVID-19 vaccine. This lower ratio of knowledge of vaccines could be associated with lower education standards in Sindh, lack of information about vaccines, and the influence of social media posting an impact on the decision-making process [19]. However, in our study, middle-aged respondents, specifically residents of big cities, diploma holders, doing private jobs were the determinate factors of higher average knowledge. Moreover, studies from Pakistan [22] and Egypt [23] have reported that older participants aged 50 to 59 years had low knowledge. In a study conducted in West India, only 35.5% of respondents had good knowledge [10], where lack of education has been one factor for lower knowledge rates.

Understanding the implications of epidemiological control of the disease and the effectiveness and progress of the vaccination drives requires a positive attitude of the general population toward the COVID-19 vaccines. Only 30.3% of respondents had a positive attitude toward COVID-19 vaccination in this study. The findings were consistent with a previous study from Sindh, Pakistan [3], which reported a similar level (34.88%) of a positive attitude toward COVID-19 among study respondents. There is a correlation between positive attitudes and acceptance of COVID-19 vaccination. This correlation may be because highly educated people remain more conscious of their health and well-being and are more involved in life events that may affect them, such as COVID-19 vaccinations. However, low-literacy rates and limited access to smartphones and the internet in rural areas of Sindh might be the contributing factors toward KAP [3].

It is estimated that 75–90% of vaccine coverage is required to achieve herd immunity [24]. It is necessary to understand the perception and sensitivity of the population towards vaccination to achieve such a higher coverage. Here we report that nearly half (48.6%) of the respondents had agreed that the COVID-19 vaccine provided by the Government of Pakistan was very safe. It has been reported that the primary concern undermining the positive attitude towards vaccination is that the new vaccines will not be safe [25, 26]. Therefore, it is suggested that disseminating factual information about the safety and effectiveness of COVID-19 vaccines is highly recommended to ensure their acceptability and coverage.

Regarding respondents’ perception of COVID-19 vaccination, about a third (33.0%) of the respondents believed that the COVID-19 vaccine might have serious side effects. The majority of respondents (42%) agreed that taking all precautions could eliminate COVID-19 without a COVID-19 vaccine. Additionally, around one-fifth of respondents (19.4%) believed that they were less likely to be infected with COVID-19 after vaccination. The fact behind misconception is based on the spread of myths about COVID-19 vaccines through social networks [27, 28]. Similarly, a study from Saudi Arabia reported that 23.4% of the participants believed that there was a chance of infection after vaccination [29]. A survey from Delhi, India, has reported that 65.7% of study participants believed that they can still be infected after vaccination which was far more than what was found in our study [30]. A Malaysian cross-sectional study reported that more than half of the participants were worried about the vaccine’s adverse effects [31]. It was also found that the majority of study respondents believed that vaccines protect themselves and others who are unvaccinated.

### Limitations of study

There are a few limitations in current research. Firstly, this study is limited only to a single province, limiting the generalizability of the results. Secondly, the study was based on an online survey; hence the response depends primarily on honesty and partly on the ability to recall. Finally, being an online survey, this study, by its nature, excludes the participants of nations who do not have access to the internet and the availability of smartphones or laptops.

## Conclusion

The Sindh population should be improved in understanding related issues to the COVID-19 vaccination process. Our research study concludes that respondents aged between 18 and 31 years, private jobholders, students, and post-graduates had sound knowledge about vaccines. The data also shows that vaccine acceptance was more among the older age group and higher qualification levels. However, there was no such impact of the profession on the acceptability of COVID-19 vaccines. Despite insufficient knowledge and a low percentage of positive attitudes, people are willing to take COVID-19 vaccines. However, the government needs to take further proactive steps to promote the information regarding the misconceptions about the COVID-19 vaccine and encourage the vulnerable groups to get vaccinated.

## Supporting information

S1 Data (XLSX)
